# Bivalent mRNA vaccine booster induces robust antibody immunity against Omicron lineages BA.2, BA.2.12.1, BA.2.75 and BA.5

**DOI:** 10.1038/s41421-022-00473-4

**Published:** 2022-10-11

**Authors:** Zhenhao Fang, Valter S. Monteiro, Anne M. Hahn, Nathan D. Grubaugh, Carolina Lucas, Sidi Chen

**Affiliations:** 1 Department of Genetics, Yale University School of Medicine, New Haven, CT USA; 2 System Biology Institute, Yale University, West Haven, CT USA; 3 Center for Cancer Systems Biology, Yale University, West Haven, CT USA; 4 Department of Immunobiology, Yale University, New Haven, CT USA; 5 Department of Epidemiology of Microbial Diseases, Yale School of Public Health, New Haven, CT USA; 6 Department of Ecology and Evolutionary Biology, Yale University, New Haven, CT USA; 7 Immunobiology Program, Yale University, New Haven, CT USA; 8 Molecular Cell Biology, Genetics, and Development Program, Yale University, New Haven, CT USA; 9 MD-PhD Program, Yale University, New Haven, CT USA; 10 Comprehensive Cancer Center, Yale University School of Medicine, New Haven, CT USA; 11 Stem Cell Center, Yale University School of Medicine, New Haven, CT USA; 12 Center for Biomedical Data Science, Yale University School of Medicine, New Haven, CT USA

**Keywords:** Biological techniques, Immunology

Dear Editor,

As the immune protection through antibodies elicited by the first booster dose wanes over time and new Omicron sublineages emerge with stronger immune evasion from humoral anti-Spike responses, the need for variant-adapted coronavirus disease 2019 (COVID-19) vaccine boosters is increasingly imminent. On June 28, vaccine advisory committee of the US Food and Drug Administration (FDA) voted in favor of updating COVID-19 vaccine booster to add an Omicron component. However, the rapid displacement of dominant Omicron lineages (from BA.1 to BA.2, then BA.2.12.1 and now BA.4, BA.5, and in some areas BA.2.75) makes it difficult to anticipate future COVID-19 vaccine targets while maintaining potency against circulating variants^1^. Each former dominant Omicron lineage, including BA.1, BA.2 and BA.2.12.1, have been replaced in a span of less than 3 months^2,3^. Reinfection or vaccine breakthrough infection caused by a new dominant variant is not uncommon due to its strong immune evasion^4,5^, which complicates the redesign of new COVID-19 boosters given the short time window of each Omicron wave and the lead time between design, validation, and deployment of new boosters.

It is a crucial question to ask which variant-based antigen(s) to use in the next generation COVID-19 boosters in order to elicit potent and broad response to past, present and emerging variants. At the time we initiated this study, the then-dominant subvariant BA.2 was gradually replaced by BA.2.12.1, BA.4, and BA.5. The L452Q/R substitutions in BA.2.12.1 and BA.4/5 are located at the receptor binding region (RBD) and ACE2 interface, and therefore associated with neutralizing antibody escape (Fig. 1a, b)^6^, with L452R detected in previous variants, including Delta, highlighting similar evolutionary trajectories in various independent variants. Omicron BA.2.75 has quickly become local dominant in some regions of India (e.g. Karnakata) in the presence of BA.5 and was found more resistant to neutralization by polyclonal sera than BA.2^7^.

**Fig. 1:**
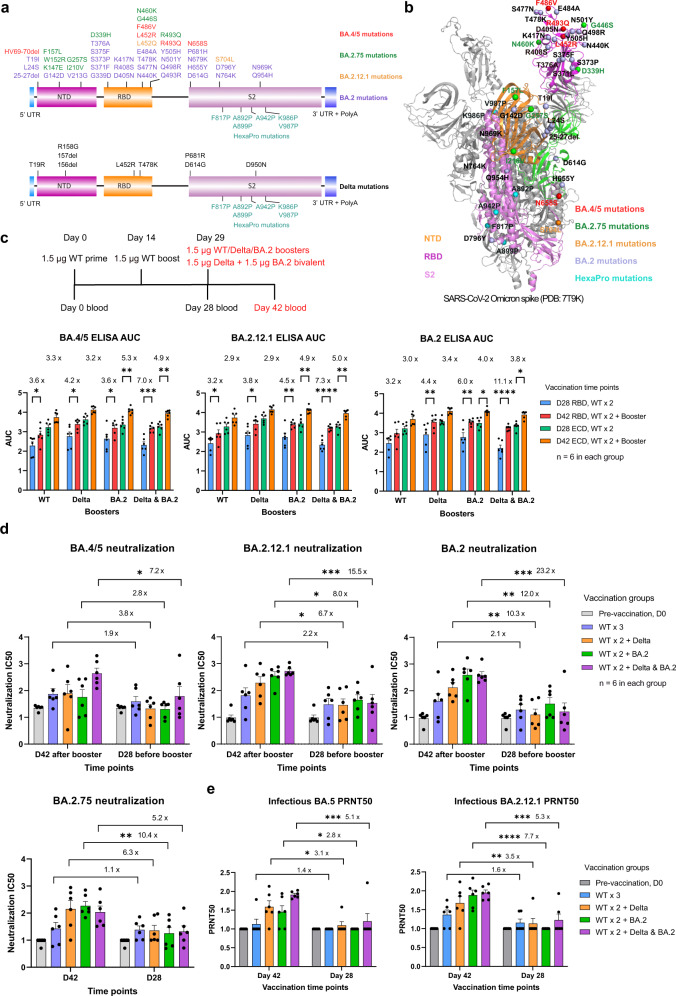
Potent antibody response to Omicron BA.2, BA.2.12.1, BA.2.75, and BA.5 subvariants by Omicron BA.2 and Delta bivalent LNP-mRNA. **a** Vaccine design of Omicron BA.2 and Delta variant-specific LNP-mRNA based on BA.2 and Delta spike mutations. Unique spike mutations on BA.2.12.1, BA.2.75, and BA.5 (not included in LNP-mRNA) are colored in orange, green, and red. **b** Distribution of BA.2 (purple), BA.2.12.1(orange), BA.2.75 (green), and BA.5 (red) mutations in one protomer of Omicron spike trimer (PDB: 7T9K). **c** Delta- and BA.2-specific monovalent or bivalent LNP-mRNA boosters improved antibody response of WT-vaccinated mice to Omicron BA.2, BA.2.12.1, and BA.4/5 subvariants. Comparison of binding antibody titers against BA.2, BA.2.12.1, and BA.4/5 spike RBD and ECD before (D28) and after (D42) receiving 1.5 µg WT-, Delta-, BA.2-specific monovalent or bivalent (1.5 µg Delta + 1.5 µg BA.2) LNP-mRNA boosters. Antibody titers were quantified by area under curves (AUC) of ELISA response curves in Supplementary Figs. S1 and S2. Blood samples were collected in mice immunized with two doses of 1.5 µg WT LNP-mRNA followed by 1.5 µg WT-, Delta-, BA.2-specific monovalent or Delta & BA.2 bivalent boosters (*n* = 6 in each group). **d** Neutralization of Omicron BA.2, BA.2.12.1, BA.2.75, and BA.5 pseudovirus by plasma of mice before (D28) and after (D42) vaccination with WT-, Delta-, BA.2-specific monovalent or Delta & BA.2 bivalent boosters. Six samples collected on day 0 were included and compared to both D28 and D42 datasets. **e** Neutralization of Omicron BA.2.12.1 and BA.5 authentic virus by plasma of mice before (D28) and after (D42) vaccination with WT-, Delta-, BA.2-specific monovalent or Delta & BA.2 bivalent boosters. Six samples collected on day 0 were included and compared to both D28 and D42 datasets. The authentic virus neutralization assay was blinded. Titer ratios before and after receiving boosters (D42/D28 ratios) were shown in Fig. 1c‒e. Titers in Fig. **d**, **e** are log_10_ transformed. Statistical analysis details can be found in supplementary methods.

Bivalent vaccine candidates have recently gained traction due to the concept of direct targeting of two variants, which may also induce broader immunity against other variants. Bivalent vaccine candidates have been under active clinical testing such as Modern’s mRNA-1273.214, which is an equal mixture of two spike-encoding mRNAs targeting ancestral SARS-CoV-2 and Omicron BA.1 (B.1.1.529), demonstrating the importance and the clinical relevance of bivalent vaccination. In light of this mix of virus genetics (Fig. 1a, b), we asked if mRNA vaccine candidates based on antigens of a circulating variant (BA.2) and/or former dominant variant (Delta) can mediate broad antibody response to emerging variants such as BA.2.12.1, BA.2.75, BA.4, or BA.5. It is worth to explore in this direction for a few reasons. The lead time of combining boosters adapted to dominant and former dominant variants will be shorter than predicting and developing boosters targeting new variants. In addition, because of the rapid displacement of circulating variants, the mismatch between the strain used for updated boosters and emerging strain may always exists. How to elicit broad response to emerging variants using existing variant antigens is an inevitable question to answer when redesigning updated COVID-19 boosters.

To answer this question, we compared the antibody response elicited by ancestral (wild type, WT), Delta, or BA.2 spike-based monovalent or Delta & BA.2 bivalent mRNA boosters against Omicron BA.2, BA.2.12.1, BA.2.75, and BA.4/5 Spike proteins. Mice were pre-immunized with two doses of 1.5 µg WT lipid nanoparticle mRNA (LNP-mRNA), followed by a booster dose with a 1.5 µg monovalent, or bivalent (1.5 µg Delta + 1.5 µg BA.2) immunization shot. All three monovalent and one bivalent booster elevated Omicron binding and neutralizing antibody titers to various degrees as indicated by ELISA and pseudovirus neutralization assay (Fig. 1c‒e; Supplementary Figs. S1‒S5, Table S1), exemplifying the benefit of receiving WT or variant-adapted booster shots against circulating emerging variants. The BA.2, BA.2.12.1, BA.2.75, and BA.5 pseudoviruses were quantified and normalized to ensure similar infection rate (Supplementary Figs. S6, S7c). Booster-associated titer ratios quantify the booster’s effect on antibody titers and were shown in each bar graph as post-booster titer on day 42 over pre-booster titer on day 28. Its dynamic range was greater in neutralization assay (ratio ranges from 1 to 23) than in ELISA (ratio ranges from 2 to 11).

Before boosters’ immunization, 24 mice in four groups received the same treatment, two doses of WT LNP-mRNA, and showed little or no significant difference in binding and neutralizing antibody titers measured on day 0 and day 28 (Supplementary Figs. S4‒S7, S8a). A minimal increase in Omicron neutralizing antibody titers was observed from mice immunized with two doses of WT LNP-mRNA (Supplementary Fig. S8b). This titer increase by WT LNP-mRNA was lowest in neutralization assay of BA.4/5 (~40% increase) as compared to BA.2.12.1, BA.2.75, and BA.2, consistent with the fact that BA.4/5 has stronger evasion of existing antibody therapeutics or vaccine-induced immunity^6^. On day 42 (2 weeks post booster), the binding as well as neutralizing titers of mice that received WT booster were lower compared to those of mice that received variant booster (Supplementary Figs. S4, S8a). These data highlight the advantage of variant-adapted boosters administration, which is consistent with our previous reports^8–10^. Interestingly, compared to the neutralizing titers against BA.2 and BA.2.12.1, BA.2 monovalent but not Delta & BA.2 bivalent booster suffered a loss of BA.4/5 pseudovirus and authentic virus neutralizing titers (Supplementary Fig. S8c, d). Collectively these indicate a broader activity of bivalent booster and strong neutralization escape of Omicron BA.4 or BA.5 even in the BA.2 mRNA-boosted individuals. In addition, RBD- and Ectodomain (ECD)- binding antibody titers directly correlated and showed distinct linear regression models between day 28 and day 42 in WT, Delta (right panel in Supplementary Fig. S5) as well as Omicron antigen datasets (left panel). The upper right shift of day 42 linear segment suggested a titer increase by boosters while the lower left shift in Omicron antigen dataset was associated with antibody evasion of Omicron epitopes.

The boosting effect of Delta- and BA.2-specific monovalent or bivalent LNP-mRNAs is universally higher than that of WT LNP-mRNA, which only modestly increased antibody titer (statistically insignificant, increase by ≤1 fold, fold change = ratio ‒ 1) in neutralization assays of Omicron BA.5, BA.2.12.1, BA.2.75, and BA.2 pseudovirus and authentic virus (Fig. 1d, e). The Delta & BA.2 bivalent booster showed superior performance of enhancing binding and neutralizing titers than either monovalent counterparts in neutralization of Omicron BA.2, BA.2.12.1, BA.4 or BA.5 pseudovirus and infectious virus, but not in neutralization of BA.2.75 pseudovirus. The bivalent booster-associated titer ratios were 23, 16, 5, and 7 fold for neutralization of BA.2, BA.2.12.1, BA.2.75, and BA.4/5 pseudovirus, respectively, while Delta/BA.2 monovalent booster ratios were 10/12, 7/8, 6/10, 4/3 respectively. The linear regression models of neutralizing and binding titers showed a trend of correlation (Supplementary Fig. S9). The neutralization titers measured on day 42 by pseudovirus and authentic virus assays were well correlated and the authentic virus titers tend to be lower (Supplementary Fig. S10).

To sum up, our data delivered a few clear messages regarding the potency of boosters against Omicron: (1) either WT or variant, monovalent or bivalent boosters can improve antibody response to Omicron BA.2, BA.2.12.1, BA.2.75 and BA.4/5, demonstrating the benefit and necessity of receiving booster shots; (2) the variant boosters with closer antigenic distance to circulating variant perform universally better than WT booster; (3) compared to monovalent booster, bivalent booster combining two genetically distant variants, Delta & BA.2 showed broader and numerically stronger antibody response to Omicron BA.2, BA.2.12.1 and BA.4/5 subvariants, but not BA.2.75. Taken together, these data provide pre-clinical evidence and rationale for developing bivalent or multi-valent variant-targeted COVID-19 boosters.

## Supplementary information

merged supplementary pdf

Supplementary Table S1
