# Supplementary material for: Bivalent mRNA vaccine booster induces robust antibody immunity against Omicron lineages BA.2, BA.2.12.1, BA.2.75 and BA.5
Source: Cell Discov. 2022 Oct 11;8:108. doi: 10.1038/s41421-022-00473-4 (PMC9552143; doi:10.1038/s41421-022-00473-4)
Supplement: Supplementary file 1 — merged supplementary pdf [file 41421_2022_473_MOESM1_ESM.pdf]

Supplementary information

Fang et al.

Bivalent mRNA vaccine booster induces robust antibody immunity against Omicron subvariants BA.2, BA.2.12.1, BA.2.75 and BA.5

Inventory of supporting information

Supplementary discussion, figures, legends and methods

Supplementary source data and statistics

Provided in excel file, “Supplementary table S1.xlsx”.

Supplementary discussions

Limitations of study

The vaccine-mediated immune response was exclusively evaluated in mice to ensure sufficient sample size and comparable genetic background. It must also be noted that, although the innate and adaptive immune system are generally conserved in mammals, mouse, NHPs and human are different species and have differences in various ways. Findings in this study is limited to preclinical mouse models, where further investigation in non-human primates (NHP) and/or potential human clinical trials are critical for future translational studies. The dosing interval between 2nd and 3rd dose in this study is 14 days, which is shorter than >6-month interval for boosters in human. A few studies demonstrated that a delayed dosing strategy improves the antibody neutralization titers against a panel of SARS-CoV-2 variants[1,2], whereas the relative titer difference between variants remains unchanged in short and extended dosing schemes. Some of antibody titer comparisons showed a trend of difference, but did not reach statistical significance due to variations and limited sample size. The insignificant results lower confidence of making related conclusions and should be interpreted together with results from companion assays to increase reliability of conclusions (ELISA, pseudovirus and authentic virus neutralization assays). In addition, the limited case number of BA.2.75 in North America makes it difficult to isolate this BA.2.75 strain and perform BA.2.75 authentic virus neutralization assay. The pseudovirus neutralization titers were correlated with authentic virus neutralization titers, but were often higher than authentic virus titers (overestimate the titers, **Supplementary Fig. 10**).

Additional background introduction

Omicron BA.4 and BA.5 lineages are thought to have emerged together with BA.1 and BA.2 and were first found in early 2022 in samples from Southern Africa[3]. These Omicron lineages quickly replaced its predecessors in populations with high previous antigen exposure from vaccination or infection of past variants. Compared to BA.2 spike, BA.2.12.1 contains two additional amino acid substitutions (L452Q and S704L) while BA.4 and BA.5 spikes are identical with four consistent substitutions (Del69-70, L452R, F486V, R493Q) plus one substitution (N658S) detected in earlier sequences. Compared to BA.2 spike sequence, BA.2.75 carries 9 additional amino acid substitutions, including K147E, W152R, F157L, I210V and G257S on N-terminal domain, and D339H, G446S, N460K and R493Q on the spike RBD region.

Supplementary figures and legends

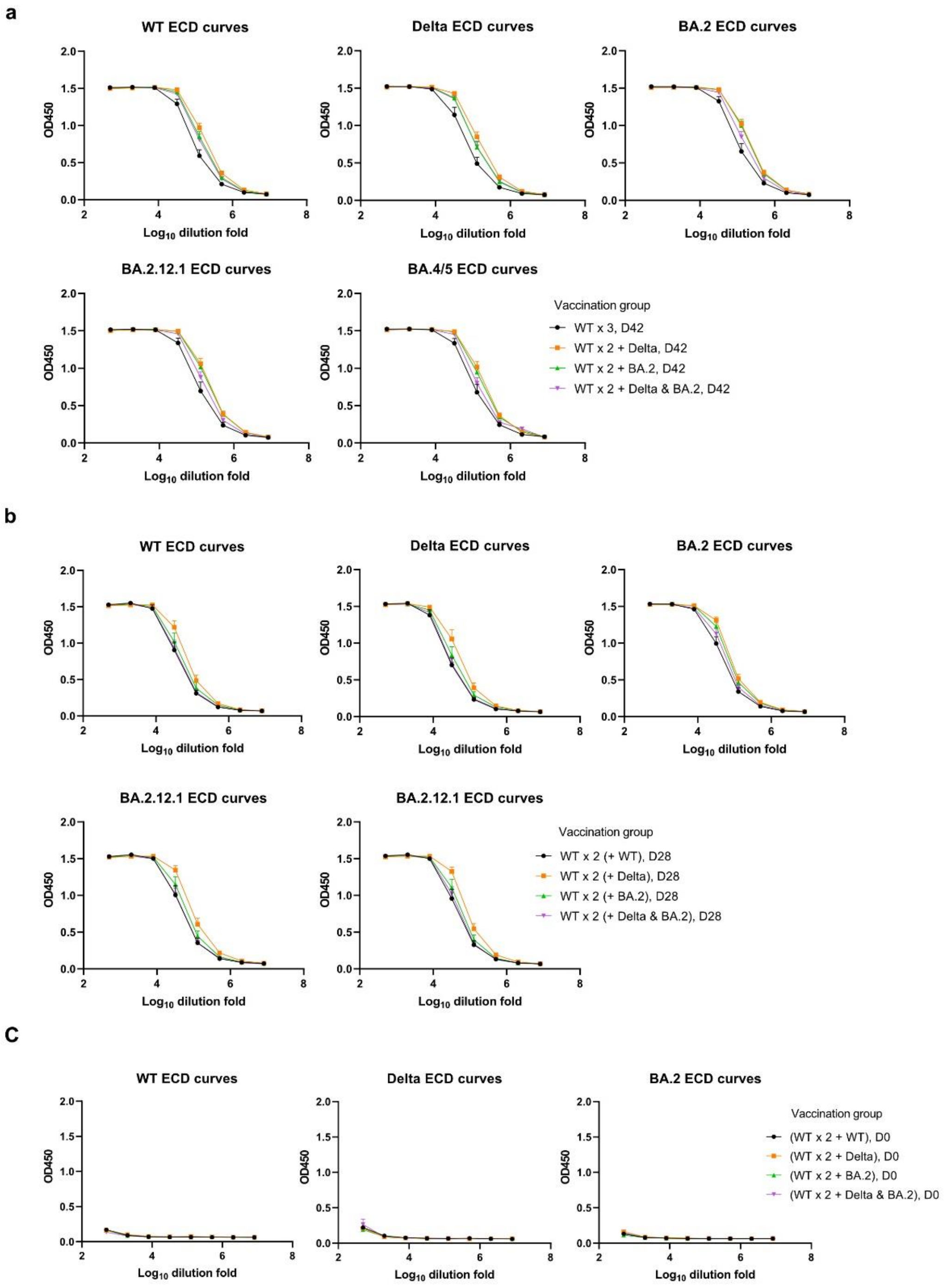

Supplementary Figure S1. Plasma dilution-dependent ELISA response curves against WT, Delta, BA.2, BA.2.12.1 and BA4/5 spike ECDs. Plasma samples were collected at day 42 (a), day 28 (b) and day 0 (c) from mice immunized with WT Delta, BA.2 specific monovalent or bivalent LNP-mRNA boosters.

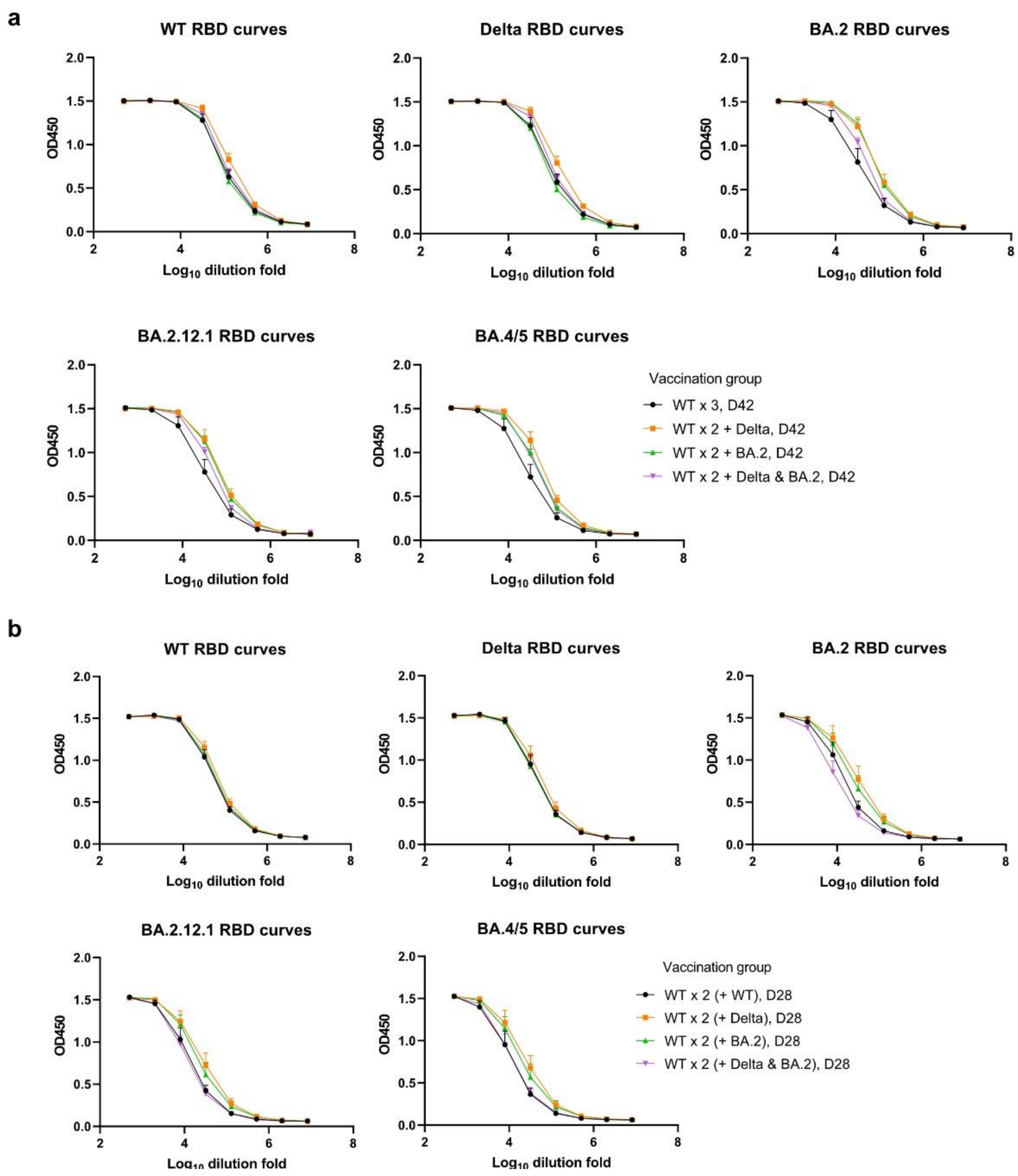

Supplementary Figure S2. Plasma dilution-dependent ELISA response curves against WT, Delta, BA.2, BA.2.12.1 and BA4/5 spike RBDs. Plasma samples were collected at day 42 (a) and day 28 (b) from mice immunized with WT Delta, BA.2 specific monovalent or bivalent LNP-mRNA boosters.

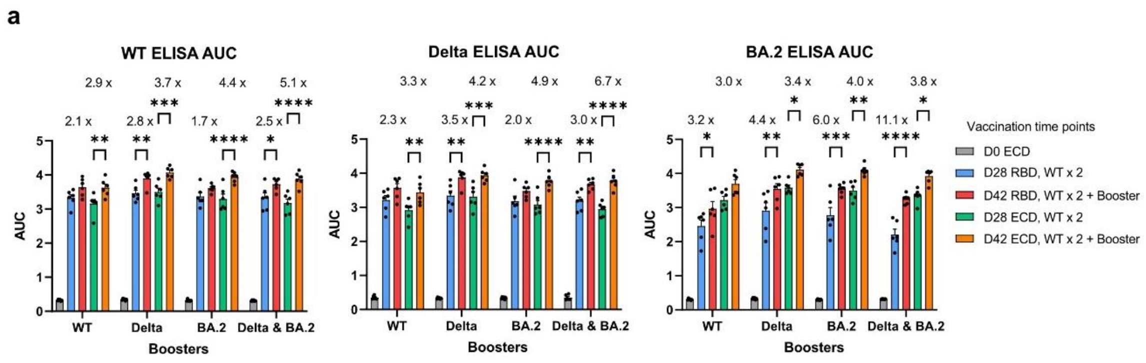

Supplementary Figure S3. **Comparison of binding antibody titers against WT (left), Delta (Mid) and BA.2 (Right) spike RBD and ECD before (D0 and D28) and after (D42) receiving 1.5 µg WT, Delta, BA.2 specific monovalent or bivalent (1.5 µg Delta + 1.5 µg BA.2) LNP-mRNA boosters (n = 6)**. Antibody titers were quantified by area under curves (AUC) of ELISA response curves in Figure S1 and S2. The comparison with day 0 samples and insignificant comparison were not shown.

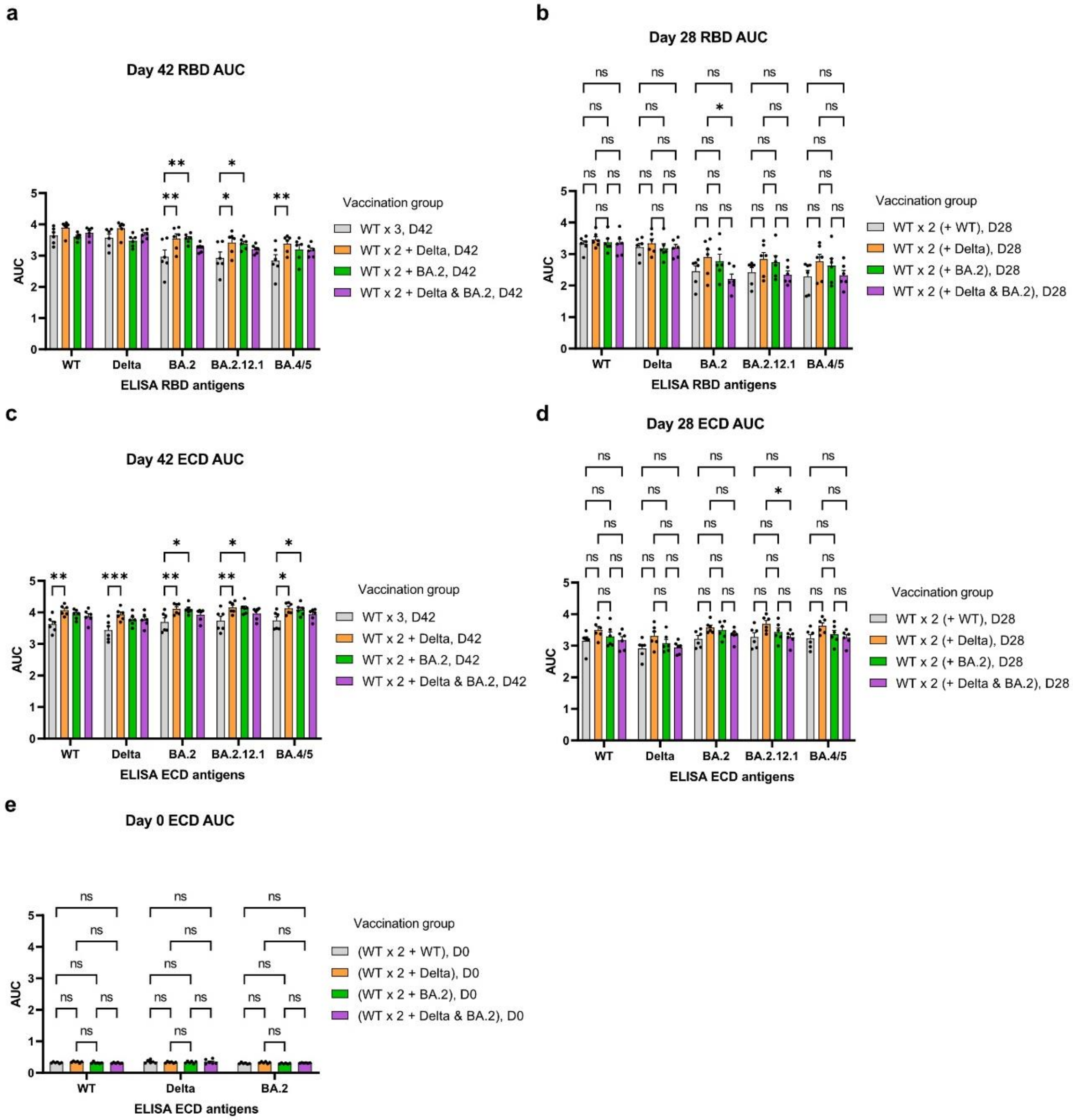

Supplementary Figure S4. Comparison of ELISA antibody titers of plasma samples collected on day 0, day 28 and day 42.

a-b, ELISA antibody titers against WT, Delta, BA.2, BA.2.12.1 and BA.4/5 spike RBDs before (D28, b) and after (D42, a) receiving 1.5 µg WT, Delta, BA.2 specific monovalent or bivalent (1.5 µg Delta + 1.5 µg BA.2) LNP-mRNA boosters.

c-e, ELISA antibody titers against WT, Delta, BA.2, BA.2.12.1 and BA.4/5 spike ECDs by plasma samples collected on (D42, c; D28, d; D0, e).

Antibody titers were quantified by area under curves (AUC) of ELISA response curves in Figure S1 and S2.

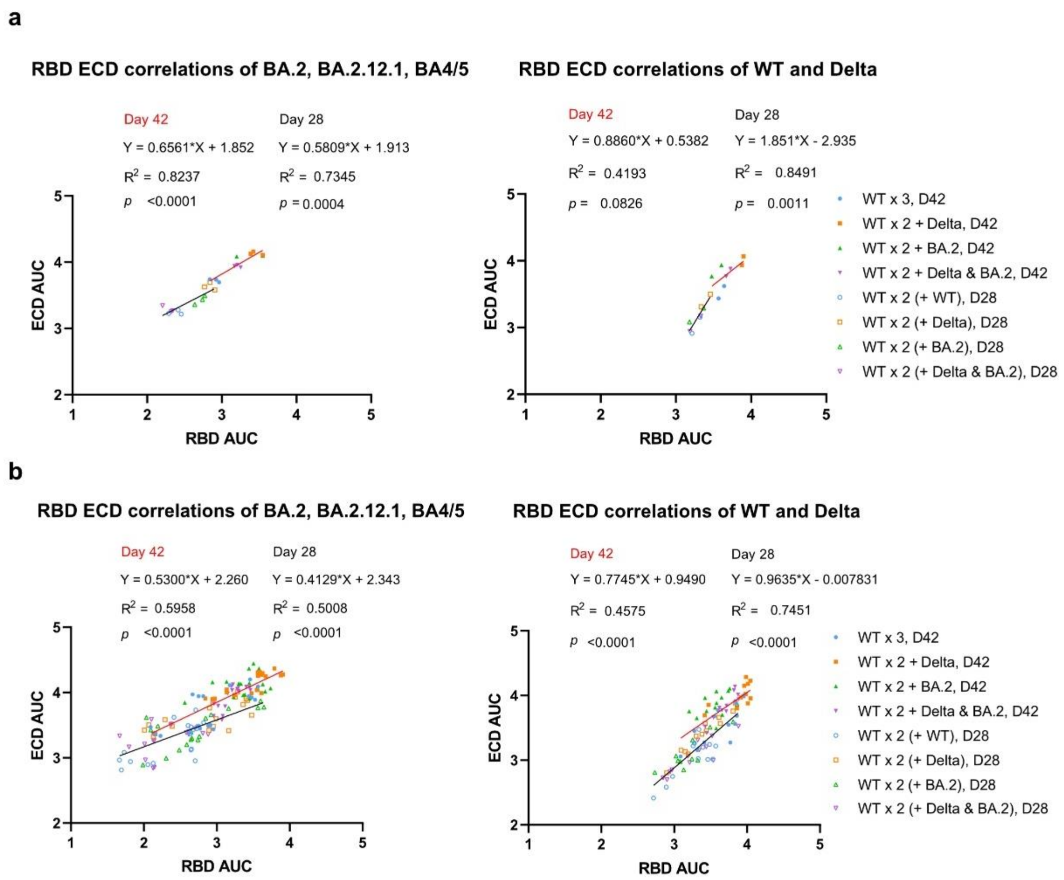

Supplementary Figure S5. Correlation of antibody titers against RBD and ECD of five spike antigens in ELISA. Antibody titers against ECD of Omicron BA.2, BA.2.12.1, BA.4/5 subvariants (left) or WT, Delta (right) were shown on y axis as $\log_{10}$ AUC and plotted against corresponding RBD binding antibody titers on x axis ($\log_{10}$ AUC). Titers were either shown as mean of matched vaccination group (a) or derived from individual animal (b).

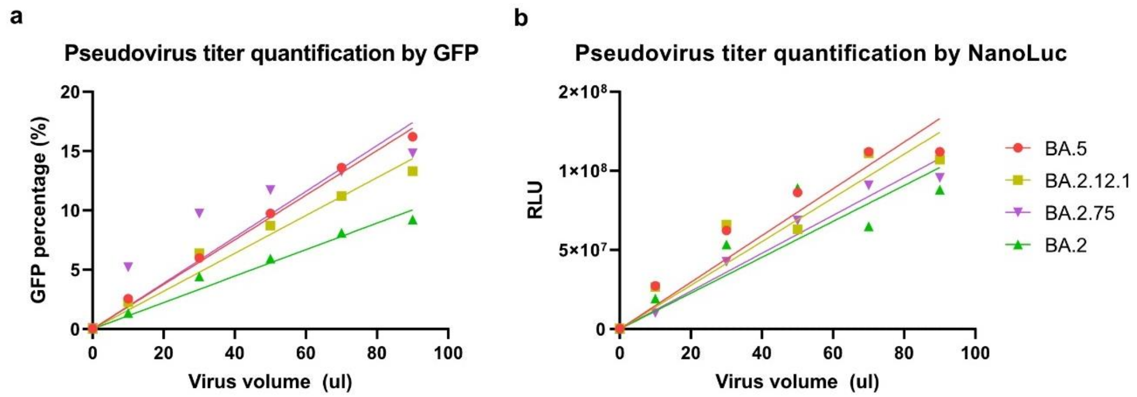

Supplementary Figure S6. Pseudovirus titer quantification by measuring GFP positive rate (a) and NanoLuciferase activity (b).

a-b, Different volume of pseudovirus was added to 293T-hACE2 cells, of which GFP positive rate (a) and Nanoluciferase activity (b) as a proxy of infection rate were measured.

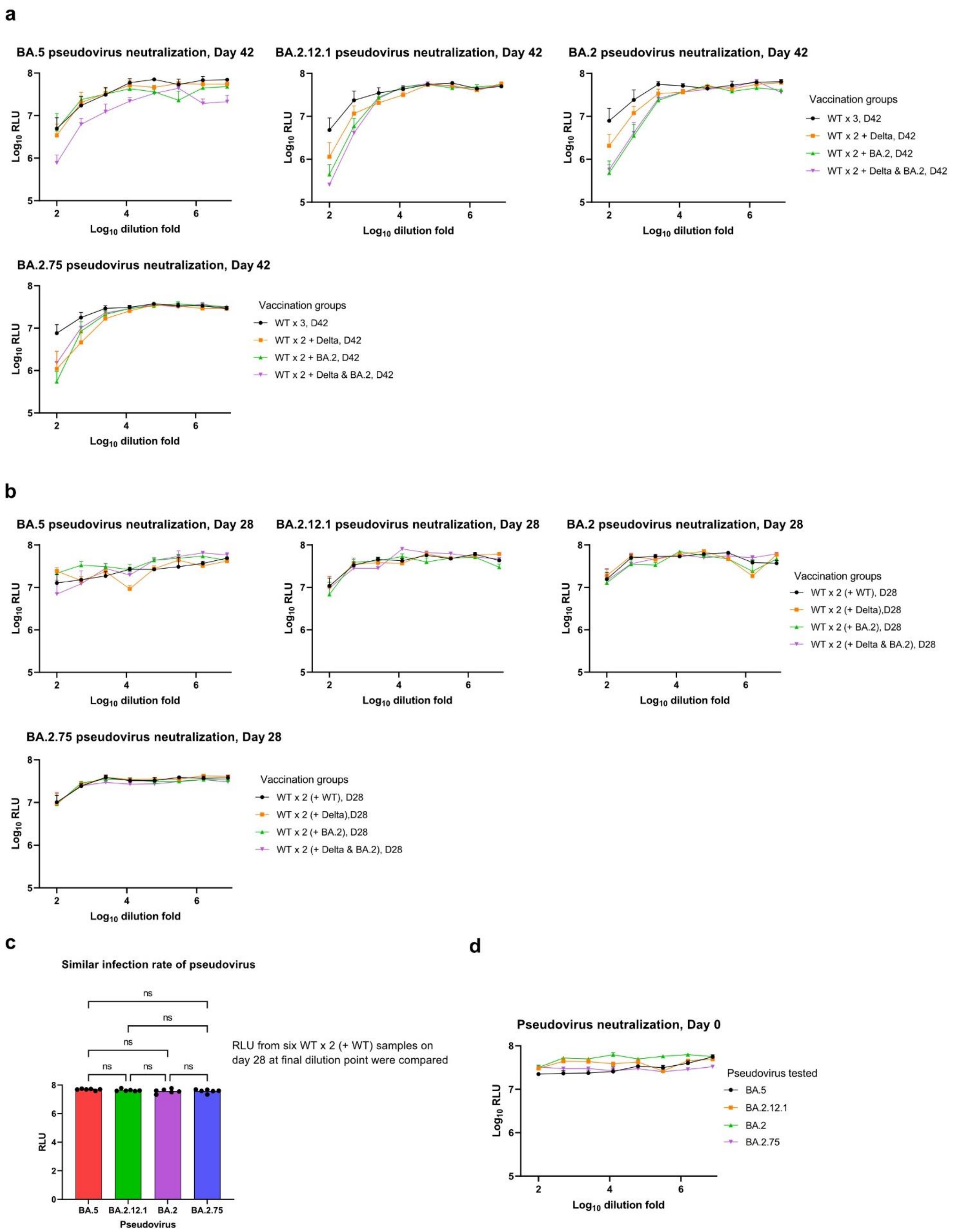
a
BA.5 pseudovirus neutralization, Day 42
BA.2.12.1 pseudovirus neutralization, Day 42
BA.2 pseudovirus neutralization, Day 42
BA.2.75 pseudovirus neutralization, Day 42
Log10 RLU
Log10 dilution fold
Vaccination groups
WT x 3, D42
WT x 2 + Delta, D42
WT x 2 + BA.2, D42
WT x 2 + Delta & BA.2, D42
b
BA.5 pseudovirus neutralization, Day 28
BA.2.12.1 pseudovirus neutralization, Day 28
BA.2 pseudovirus neutralization, Day 28
BA.2.75 pseudovirus neutralization, Day 28
Vaccination groups
WT x 2 (+ WT), D28
WT x 2 (+ Delta),D28
WT x 2 (+ BA.2), D28
WT x 2 (+ Delta & BA.2), D28
c
Similar infection rate of pseudovirus
ns
RLU from six WT x 2 (+ WT) samples on day 28 at final dilution point were compared
RLU
BA.5
BA.2.12.1
BA.2
BA.2.75
Pseudovirus
d
Pseudovirus neutralization, Day 0
Pseudovirus tested
BA.5
BA.2.12.1
BA.2
BA.2.75

Supplementary Figure S7. Neutralization titration curves of serially diluted plasma collected at indicated time points from mice vaccinated with WT, Delta, BA.2 monovalent or bivalent LNP-mRNA boosters.

a, Neutralization curves of BA.5, BA.2.12.1, BA.2.75 and BA.2 pseudovirus by samples collected on day 42 from mice immunized with 1.5 µg WT, Delta, BA.2 monovalent or bivalent LNP-mRNA boosters.

b, Neutralization curves of BA.5, BA.2.12.1, BA.2.75 and BA.2 pseudovirus by samples collected on day 28 from mice immunized with two doses of 1.5 µg WT LNP-mRNA.

c, RLUs of six WT x 2 (+ WT) samples on day 28 at the last dilution point (10^6.9 dilution) were compared among four pseudovirus neutralization assays. RLUs or infection rates of BA.5, BA.2.12.1, BA.2.75 and BA.2 pseudovirus were similar.

d, Neutralization curves of BA.5, BA.2.12.1, BA.2.75 and BA.2 pseudovirus by samples collected on day 0 from vaccination naïve mice.

The $\log_{10}$ relative light unit (RLU) measured by NanoLuc luciferase assay were shown as mean ± s.e.m. and plotted against serial log10-transformed sample dilution points.

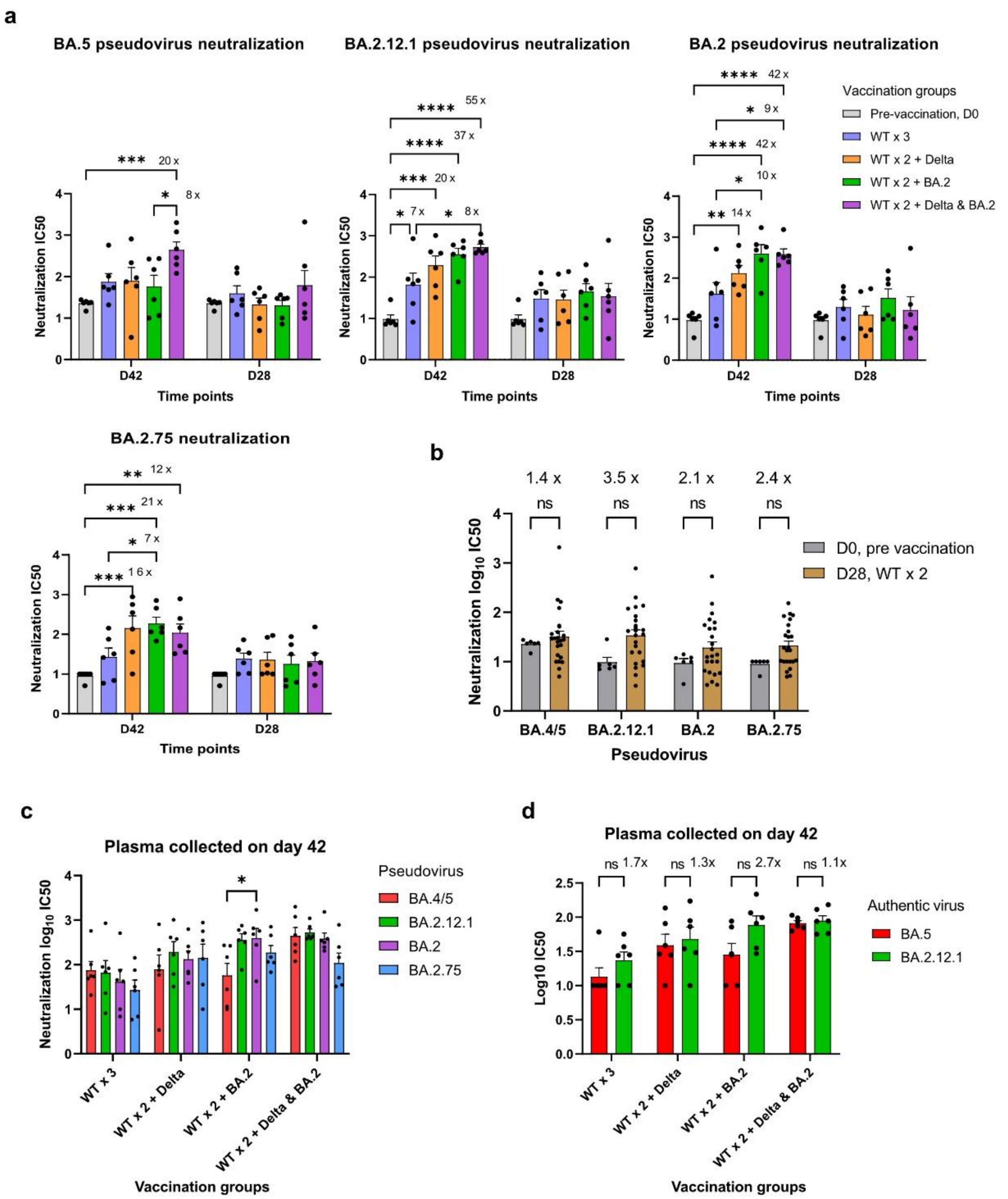

Supplementary Figure S8. Statistical comparison of neutralizing titers of plasma samples from different vaccination groups at same time point (a, c, d) or against different Omicron subvariant pseudoviruses at matched time points (b).

a, Omicron BA.2 (right), BA.2.12.1 (mid), BA.5 (left) and BA.2.75 (second row) pseudovirus neutralization by plasma of mice before (D28) and after (D42) vaccinated with WT, Delta, BA.2 specific monovalent or Delta & BA.2 bivalent boosters. Six samples collected on day 0 were included and compared to both D28 and D42 datasets.

b, BA.4/5, BA.2.12.1, BA.2.75 and BA.2 neutralizing antibody titers from samples collected on day 0 and day 28 (WT x 2) were compared.

c, BA.4/5, BA.2.12.1, BA.2.75 and BA.2 pseudovirus neutralizing antibody titers were compared within same vaccination groups at the same time on day 42 (post booster).

d, BA.4/5 and BA.2.12.1 authentic virus neutralizing antibody titers were compared within same vaccination groups at the same time on day 42 (post booster).

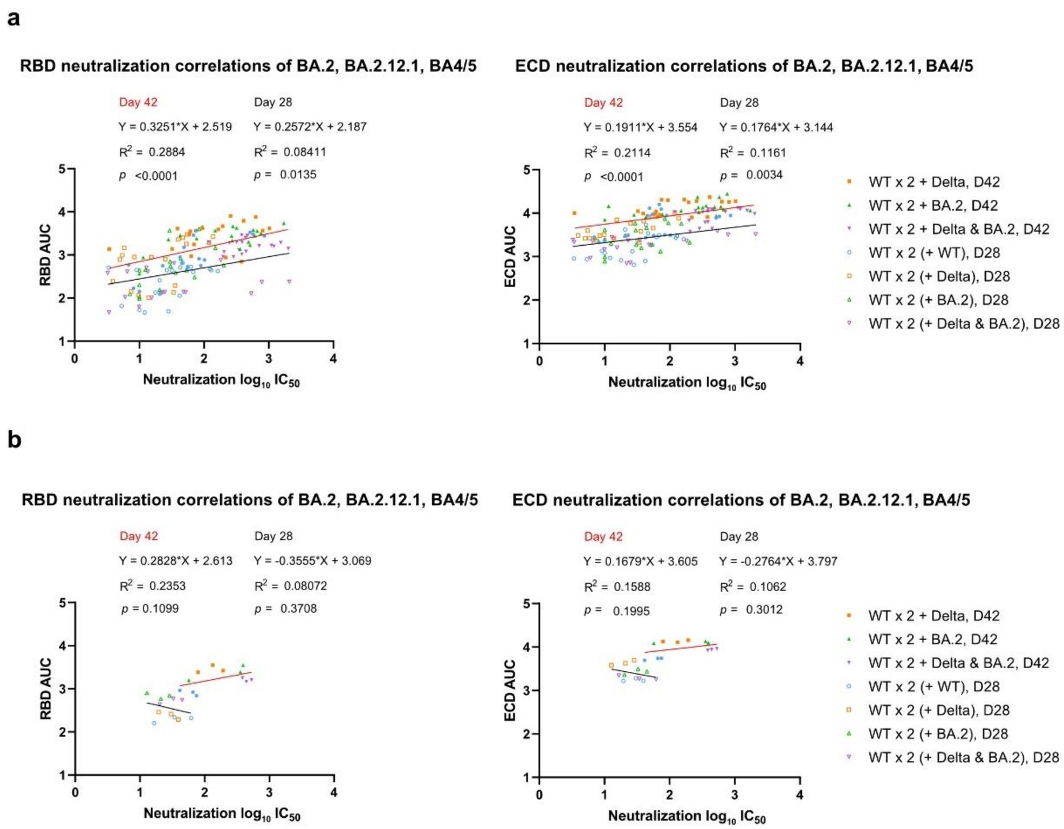

Supplementary Figure S9. Correlation of antibody titers measured by pseudovirus neutralization and ELISA. Antibody titers determined by pseudovirus neutralization assay were shown on x axis as log_{10} IC50 and plotted against ELISA binding antibody titers (log10 AUC) measured by RBD (left) or ECD (right) spike antigens on y axis. Titer values were either derived from mean of matched vaccination group (b) or individual animals (a).

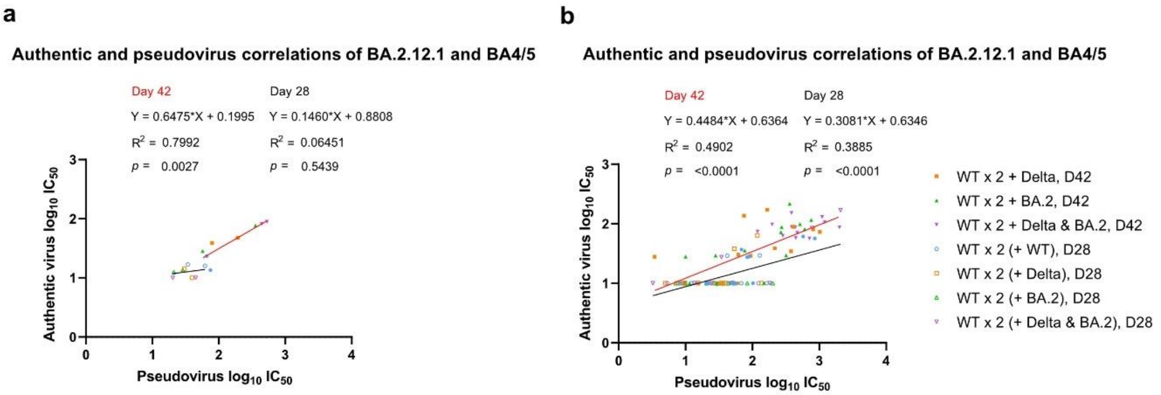

Supplementary Figure S10. Correlation of antibody titers measured by pseudovirus and authentic virus neutralization. Antibody titers determined by pseudovirus and authentic virus neutralization assays were shown on x axis as pseudovirus $\log_{10}$ IC50 and plotted against authentic virus neutralizing titers (y axis). Titer values were either derived from mean of matched vaccination group (a) or individual animals (b).

Methods

Institutional approval

All animal work was performed under the guidelines of Yale University Institutional Animal Care and Use Committee (IACUC) with approved protocols (Chen 2020-20358; Chen 2021-20068). All recombinant DNA (rDNA) and biosafety work were performed under the guidelines of Yale Environment, Health and Safety (EHS) Committee with approved protocols (Chen 18–45, 20–18, and 20–26). The Yale Human Research Protection Program Institutional Review Board determined that the sequencing and generating a virus isolate from de-identified remnant COVID-19 clinical samples conducted in this study were not research involving human participants (IRB protocol ID 2000028599).

Molecular cloning and mRNA preparation

The WT and Delta spike plasmids were cloned in our previous study[4,5]. BA.2 spike plasmid was cloned based on the isolate sequencing data in GISAID EpiCoV (EPI_ISL_6795834.2)[6]. WT, Delta and BA.2 spike plasmids were linearized by restriction enzymes and transcribed to mRNA by in vitro T7 RNA polymerase (NEB, Cat # E2060S) as previously described[5].

Cell culture

hACE2-293FT and 293T cells were cultured in Dulbecco's minimal essential medium (DMEM, Fisher) supplemented with 10% fetal bovine serum (Hyclone) and penicillin (100 U/ml)-streptomycin (100 ug/ml). Cells were split ever other day at a 1:4 ratio when confluency is over 90%.

Lipid nanoparticle mRNA preparation

In brief, lipids mixture was solubilized in ethanol and mixed with spike mRNA in pH 5.2 sodium acetate buffer. The lipids mixture contains ALC-0315, ALC-0159, DSPC and cholesterol at a mixing ratio of 46.3:1.6:9.4:42.7 as previously described[5]. The mRNA encapsulated by LNP (LNP-mRNA) was then buffer exchanged to PBS using 100kDa Amicon filter (Macrosep Centrifugal Devices 100K, 89131-992). The size distribution of LNP-mRNA was evaluated by dynamic light scatter (DynaPro NanoStar, Wyatt, WDPN-06).

The Quant-iT™ RiboGreen™ (Thermo Fisher) RNA Assay was applied to determine encapsulation rate and mRNA amount.

Animal vaccination

Animal immunization was performed on 16-18 weeks female C57BL/6Ncr mice purchased from Charles River. Mice were vaccinated with two doses of 1.5 µg WT LNP-mRNA on day 0 and day 14 followed by 1.5 µg WT, Delta, Omicron BA.2 monovalent booster or Delta & BA.2 bivalent booster on day 29. The plasma samples were isolated from blood which was collected before vaccination on day 0, two weeks after WT boost on day 28 and two weeks after monovalent or bivalent boosters on day 42.

ELISA and Neutralization assay

The binding and neutralizing antibody titers were determined by ELISA and pseudovirus neutralization assay as previously described[5]. NanoGlo luciferase assay system (Promega N1120) was applied to determine the pseudovirus infection level in hACE2-293FT cells. The ELISA antigens including RBDs of WT (Sino 40592-V08B), Delta(Sino 40592-V08H90), Omicron BA.2(Acro SPD-C522g-100ug), BA.2.12.1(Acro SPD-C522q-100ug) and BA.4/5(Acro SPD-C522r-100ug) were purchased from Sino Biological and AcroBiosystems. The ELISA ECD antigens including WT (Sino 40589-V08B1), Delta (Sino 40589-V08B16), Omicron BA.2 (Acro SPN-C5223-50ug), BA.2.12.1 (Acro SPN-C522d-50ug) and BA.4/5 (SPN-C5229-50ug) were purchased from Sino Biological and AcroBiosystems. The pseudovirus plasmids of spike without HexaPro mutations were generated based on the WT plasmid which was a gift from Dr. Bieniasz's lab. The pseudovirus titers were quantified by measuring the GFP positive rate and NanoLuc activity when different volume of virus supernatant was added (**Supplementary Fig. S6**). Because of better linear regression fitting, the linear model from GFP dataset was used to normalize all pseudoviruses to ensure similar infection rate of different pseudovirueses. After model fitting, the derived Log_{10} IC50 value in pseudovirus neutralization less than 0.5 is converted to 1.

Authentic virus neutralization

The mouse plasma was heat inactivated prior to infectious virus neutralization assay in order to remove complements and other potential neutralizing agents. Mouse plasma samples were serially diluted, then incubated with SARS-CoV-2 Omicron BA.2.12.1 or BA.5 infectious virus for 1 h at 37 °C. The Omicron BA.2.12.1 and BA.5 authentic viruses were isolated from nasopharyngeal specimens and sequenced as part of the Yale SARS-CoV-2 Genomic Surveillance Initiative's weekly surveillance Program in Connecticut. After coincubation, plasma/virus mixture was added to Vero-E6 cells overexpressing ACE2/TMPRSS2. Cell viability was measured at 3dpi or 5dpi using CellTiter Glo[6].

Statistics

For grouped bar statistical analysis in Figure 1 and S3-S4, Individual dot in dot-bar plots represents value from each mouse and is shown as mean ± s.e.m.. To assess statistical significance, two-way ANOVA with Tukey's or Šídák's multiple comparisons test was used. Statistical significance labels: * $p < 0.05$; ** $p < 0.01$; *** $p < 0.001$; **** $p < 0.0001$. Non significant comparisons are not shown. The statistical analysis of each comparison was included in the Supplementary Table S1 excel file.

Data availability

All source data and statistical analysis are provided in this article and its supplementary excel file.

Code availability

No custom code was used in this study.

Protein sequence of WT HexaPro spike

MFVFLVLLPLVSSQCVNLTTRTQLPPAYTNSFTRGVYYPDKVFRSSVLHSTQDLFLPFFSNVTWFHAIHVSGTNGTKRFD
NPVLPFNDGVYFASTEKSNIIRGWIFGTTLDSKTQSLLIVNNATNVVIKVCEFQFCNDPFLGVYYHKNNKSWMESEFRVY
SSANNCTFEYVSQPFLMDLEGKQGNFKNLREFVFKNIDGYFKIYSKHTPINLVRDLPQGFSALEPLVDLPIGINITRFQTLL
ALHRSYLTPGDSSSGWTAGAAAYYVGYLQPRTFLLKYNENGTITDAVDCALDPLSETKCTLKSFTVEKGIYQTSNFRVQP
TESIVRFPNITNLCPFGEVFNATRFASVYAWNRKRISNCVADYSVLYNSASFSTFKCYGVSPTKLNDLCFTNVYADSFVIR
GDEVRQIAPGQTGKIADYNYKLPDDFTGCVIAWNSNNLDSKVGGNYNYLYRLFRKSNLKPFERDISTEIYQAGSTPCNG
VEGFNCYFPLQSYGFQPTNGVGYQPYRVVVLSFELLHAPATVCGPKKSTNLVKNKCVNFNFNGLTGTGVLTESNKKFLP
FQQFGRDIADTTDAVRDPQTLEILDITPCSFGGVSVITPGTNTSNQVAVLYQDVNCTEVPVAIHADQLTPTWRVYSTGS

NVFQTRAGCLIGAEHVNNSYECDIPIGAGICASYQTQTNSPGSASSVASQSIIAYTMSLGAENSVAYSNNSIAIPTNFTISVTTEILPVSMTKTSVDCTMYICGDSTECSNLLLQYGSFCTQLNRALTGIAVEQDKNTQEVFAQVKQIYKTPPIKDFGGFNFSQILPDPSKPSKRSPIEDLLFNKVTLADAGFIKQYGDCLGDIAARDLICAQKFNGLTVLPPLLTDEMIAQYTSALLAGTITSGWTFGAGPALQIPFPMQMAYRFNGIGVTQNVLYENQKLIANQFNSAIGKIQDSLSSTPSALGKLQDVVNQNAQALNTLVKQLSSNFGAISSVLNDILSRLDPPEAEVQIDRLITGRLQSLQTYVTQQLIRAAEIRASANLAATKMSECVLGQSKRVDFCGKGYHLMSFPQSAPHGVVFLHVTYVPAQEKNFTTAPAICHDGKAHFPREGVFVSNGTHWFVTQRNFYEPQIITTDNTFVSGNCDVVIGIVNNTVYDPLQPELDSFKEELDKYFKNHTSPDVDLGDISGINASVVNIQKEIDRLNEVAKNLNESLIDLQELGKYEQYIKWPWYIWLGFIAGLIAIVMVTIMLCCMTSCCSCLKGCCSCGSCCKFDEDDSEPVLKGVKLHYT*

Protein sequence of Delta HexaPro spike

MFVFLVLLPLVSSQCVNLRTRTQLPPAYTNSFTRGVYYPDKVFRSSVLHSTQDLFLPFFSNVTWFHAIHVSGTNGTKRFDNPVLPFNDGVYFASTEKSNIIRGWIFGTTLDSKTQSLLIVNNATNVVIKVCEFQFCNDPFLDVYYHKNNKSWMESGVYSSANNCTFEYVSQPFLMDLEGKQGNFKNLREFVFKNIDGYFKIYSKHTPINLVRDLPQGFSALEPLVDLPIGINITRFQTLLALHRSYLTPGDSSSGWTAGAAAYYVGYLQPRTFLLKYNENGTITDAVDCALDPLSETKCTLKSFTVEKGIYQTSNFRVQPTESIVRFPNITNLCPFGEVFNATRFASVYAWNRKRISNCVADYSVLYNSASFSTFKCYGVSPTKLNDLCFTNVYADSFVIRGDEVRQIAPGQTGKIADYNYKLPDDFTGCVIAWNSNNLDSKVGGNYNYRYRLFRKSNLKPFERDISTEIYQAGSKPCNGVEGFNCYFPLQSYGFQPTNGVGYQPYRVVVLSFELLHAPATVCGPKKSTNLVKNKCVNFNFNGLTGTGVLTESNKKFLPFQQFGRDIADTTDAVRDPQTLEILDITPCSFGGVSVITPGTNTSNQVAVLYQGVNCTEVPVAIHADQLTPTWRVYSTGSNVFQTRAGCLIGAEHVNNSYECDIPIGAGICASYQTQTNSRGSASSVASQSIIAYTMSLGAENSVAYSNNSIAIPTNFTISVTTEILPVSMTKTSVDCTMYICGDSTECSNLLLQYGSFCTQLNRALTGIAVEQDKNTQEVFAQVKQIYKTPPIKDFGGFNFSQILPDPSKPSKRSPIEDLLFNKVTLADAGFIKQYGDCLGDIAARDLICAQKFNGLTVLPPLLTDEMIAQYTSALLAGTITSGWTFGAGPALQIPFPMQMAYRFNGIGVTQNVLYENQKLIANQFNSAIGKIQDSLSSTPSALGKLQNVVNQNAQALNTLVKQLSSNFGAISSVLNDILSRLDPPEAEVQIDRLITGRLQSLQTYVTQQLIRAAEIRASANLAATKMSECVLGQSKRVDFCGKGYHLMSFPQSAPHGVVFLHVTYVPAQEKNFTTAPAICHDGKAHFPREGVFVSNGTHWFVTQRNFYEPQIITTDNTFVSGNCDVVIGIVNNTVYDPLQPELDSFKEELDKYFKNHTSPDVDLGDISGINASVVNIQKEIDRLNEVAKNLNESLIDLQELGKYEQYIKWPWYIWLGFIAGLIAIVMVTIMLCCMTSCCSCLKGCCSCGSCCKFDEDDSEPVLKGVKLHYT*

Protein sequence of BA.2 HexaPro spike

MFVFLVLLPLVSSQCVNLITRTQSYTNSFTRGVYYPDKVFRSSVLHSTQDLFLPFFSNVTWFHAIHVSGTNGTKRFDNPVLPFNDGVYFASTEKSNIIRGWIFGTTLDSKTQSLLIVNNATNVVIKVCEFQFCNDPFLDVYYHKNNKSWMESEFRVYSSANNCTFEYVSQPFLMDLEGKQGNFKNLREFVFKNIDGYFKIYSKHTPINLGRDLPQGFSALEPLVDLPIGINITRFQTLLALHRSYLTPGDSSSGWTAGAAAYYVGYLQPRTFLLKYNENGTITDAVDCALDPLSETKCTLKSFTVEKGIYQTSNFRVQPTESIVRFPNITNLCPFDEVFNATRFASVYAWNRKRISNCVADYSVLYNFAPFFAFKCYGVSPTKLNDLCFTNVYADSFVIRGNEVSQIAPGQTGNIADYNYKLPDDFTGCVIAWNSNKLDSKVGGNYNYLYRLFRKSNLKPFERDISTEIYQAGNKPCNGVAGFNCYFPLRSYGFRPTYGVGHQPYRVVVLSFELLHAPATVCGPKKSTNLVKNKCVNFNFNGLTGTGVLTESNKKFLPFQQFGRDIADTTDAVRDPQTLEILDITPCSFGGVSVITPGTNTSNQVAVLYQGVNCTEVPVAIHADQLTPTWRVYSTGSNVFQTRAGCLIGAEYVNNSYECDIPIGAGICASYQTQTKSHRRARSVASQSIIAYTMSLGAENSVAYSNNSIAIPTNFTISVTTEILPVSMTKTSVDCTMYICGDSTECSNLLLQYGSFCTQLKRALTGIAVEQDKNTQEVFAQVKQIYKTPPIKYFGGFNFSQILPDPSKPSKRSPIEDLLFNKVTLADAGFIKQYGDCLGDIAARDLICAQKFNGLTVLPPLLTDEMIAQYTSALLAGTITSGWTFGAGPALQIPFPMQMAYRFNGIGVTQNVLYENQKLIANQFNSAIGKIQDSLSSTPSALGKLQDVVNHNAQALNTLVKQLSSKFGAISSVLNDILSRLDPPEAEVQIDRLITGRLQSLQTYVTQQLIRAAEIRASANLAATKMSECVLGQSKRVDFCGKGYHL

MSFPQSAPHGVVFLHVTYVPAQEKNFTTAPAICHDGKAHFPREGVFVSNGTHWFVTQRNFYEPQIITTDNTFVSGNCD
VVIGIVNNTVYDPLQPELDSFKEELDKYFKNHTSPDVDLGDISGINASVVNIQKEIDRLNEVAKNLNESLIDLQELGKYEQY
IKWPWYIWLGFIAGLIAIVMVTIMLCCMTSCCSCLKGCCSCGSCCKFDEDDSEPVLKGVKLHYT*

References

1 Payne, R. P. *et al.* Immunogenicity of standard and extended dosing intervals of BNT162b2 mRNA vaccine. *Cell* **184**, 5699-5714 e5611, doi:10.1016/j.cell.2021.10.011 (2021).

2 Hall, V. G. *et al.* Delayed-interval BNT162b2 mRNA COVID-19 vaccination enhances humoral immunity and induces robust T cell responses. *Nat Immunol* **23**, 380-385, doi:10.1038/s41590-021-01126-6 (2022).

3 Tegally, H. *et al.* Emergence of SARS-CoV-2 Omicron lineages BA.4 and BA.5 in South Africa. *Nat Med*, doi:10.1038/s41591-022-01911-2 (2022).

4 Peng, L. *et al.* Variant-specific vaccination induces systems immune responses and potent in vivo protection against SARS-CoV-2. *Cell Rep Med* **3**, 100634, doi:10.1016/j.xcrm.2022.100634 (2022).

5 Fang, Z. *et al.* Omicron-specific mRNA vaccination alone and as a heterologous booster against SARS-CoV-2. *Nat Commun* **13**, 3250, doi:10.1038/s41467-022-30878-4 (2022).

6 Fang, Z. et al. Heterotypic vaccination responses against SARS-CoV-2 Omicron BA.2. *Cell Discov* **8**, 69 (2022)
